# Potential Antioxidant and Enzyme Inhibitory Effects of Nanoliposomal Formulation Prepared from *Salvia aramiensis* Rech. f. Extract

**DOI:** 10.3390/antiox9040293

**Published:** 2020-04-01

**Authors:** Gökçe Şeker Karatoprak, Çiğdem Yücel, Fatih Göger, Eduardo Sobarzo-Sánchez, Esra Küpeli Akkol

**Affiliations:** 1 Department of Pharmacognosy, Faculty of Pharmacy, Erciyes University, 38039 Kayseri, Turkey; gskaratoprak@erciyes.edu.tr; 2 Department of Pharmaceutical Technology, Faculty of Pharmacy, Erciyes University, 38039 Kayseri, Turkey; cyucel@erciyes.edu.tr; 3 Department of Pharmacognosy, Faculty of Pharmacy, Anadolu University, 26470 Eskişehir, Turkey; fatihgoger@anadolu.edu.tr; 4 Department of Pharmacy, Yunus Emre Vocational School, Anadolu University, 26470 Eskişehir, Turkey; 5 Instituto de Investigación e Innovación en Salud, Facultad de Ciencias de la Salud, Universidad Central de Chile, 8330507 Santiago, Chile; 6 Department of Organic Chemistry, Faculty of Pharmacy, University of Santiago de Compostela, 15782 Santiago de Compostela, Spain; 7 Faculty of Pharmacy, Department of Pharmacognosy, Gazi University, 06330 Ankara, Turkey

**Keywords:** *Salvia aramiensis*, Lamiaceae, antioxidant, enzyme, chromatography, nanoliposome

## Abstract

*Salvia aramiensis* Rech. f. is a species that grows only in Hatay, Turkey and is used as a traditional stomachic tea. Neither the chemical composition nor the potential bioactivity of the plant has been investigated before. Antioxidant activity (1,1-Diphenyl-2-picrylhydrazyl Radical (DPPH^●^) and 2,2’-Azino-bis (3-ethylbenzothiazoline-6-sulfonic acid (ABTS^+●^) radical scavenging and β-carotene/linoleic acid co-oxidation) of 70% methanol, 70% ethanol extracts, and 2% infusion obtained from *S. aramiensis* aerial parts were determined. The effect of 70% methanol extract on collagenase and elastase enzyme inhibition and its chemical composition via chromatographic methods (LC-MS/MS and HPLC) were analyzed. Nanoliposomes were developed with 70% methanol extract, were characterized, and were evaluated. The key parameters for the most active 70% methanol extract included the following DPPH^•^EC_50_: 28.4 µg/mL, Trolox equivalent antioxidant capacity (TEAC)/ABTS: 1.77 ± 0.09 mmol/L/Trolox. Furthermore 70% methanol extract showed more than 50% inhibition on collagenase and elastase enzymes at all the concentrations. The main component of the extract, rich in phenolic compounds, has been identified as rosmarinic acid; 83.7 µg/mL extract was released from the nanoliposomal formulation. The extract and its formulation are found to be nontoxic on the L929 fibroblast cell line. This study successfully developed a long-term antioxidant and enzyme inhibitory formulation containing *S. aramiensis*, which has been used safely among the public for years.

## 1. Introduction

The family Lamiaceae has 236 genera and about 6900 to 7200 species. *Salvia* (~900 species) is one of the largest genera of Lamiaceae [1]. The name derives from the Latin word “salveo”, that means “to save, to heal” [2]. *Salvia* species have been used as tea since ancient times to prevent colds, coughs, nervous exhaustion, stomach and abdominal pain, pharyngitis, inflammation of the mouth, inflammation of the gums, excessive sweating, and increased lactation [3,4,5]. Many studies on *Salvia* species have shown that plants, extracts, and essential oils possess biological activities such as antiseptic, antifungal, antibacterial, antiviral, analgesic, antispasmodic, antioxidant, astringent, hallucinogenic, central nervous system depressant, anticancer, cardiovascular, antidiabetic, and insecticidal activities [6]. As a result of the phytochemical studies, it has been learned that these plants are rich in flavonoids, phenolic compounds, as well as diterpenes and triterpenes [7,8,9]. These compounds show a natural antioxidant property by stopping or inhibiting the reactions caused by free radicals [10,11].

*Salvia aramiensis* Rech. f. grown in *Pinus brutia* woodlands, rocky places, and limestones in Hatay (Turkey) province, are perennial, evergreen, and subshrub with woody stems [12]. Its flowers and leafy branches are used as stomachic herbal tea [13]. The literature has focused on *S. aramiensis* essential oil composition and its antioxidant and antimicrobial activity [14]. According to literature, *S. aramiensis* essential oil is a potent antimicrobial and antioxidant agent [2,6,15]. To date, the biological activity and chemical composition of *S. aramiensis* have not been investigated.

Despite extensive research into the discovery of new collagenase, elastase, and hyaluronidase enzyme-inhibiting compounds of both synthetic and natural origin, it is still a major basis for recent inhibitors of these enzymes due to the side effects or low efficacy of existing enzymes. Also, the current number of these enzyme inhibitors is very limited, and recent inhibitors are in demand mainly in the cosmetic and pharmaceutical industry (wound healing) [16]. The dermis, the middle layer of the skin, consists of elastin and collagen, the main component of the connective tissue. These proteins are responsible for the resistance and elasticity of the skin and are destroyed as a result of the formation of free radicals and the induction of elastase and collagenase enzymes [17]. Collagen and elastin also play an important role in the wound-healing process. Inhibition of collagenase activity retards the progression of formation of pre-collagen fibers [18]. Overproduced elastase enzyme accelerates the degradation of the surrounding healthy tissue around the wound by catalyzing this protein [18,19]. Reactive oxygen species (ROS) are one of the factors that trigger skin aging and that delay the wound healing process by causing oxidative damage of skin lipids, proteins, and DNA [20,21]. To increase the antiaging effect, to prevent loss of skin elasticity, and to accelerate wound healing, it is essential to find inhibitors of elastase and collagenase enzymes, which have a radical scavenging feature.

Encapsulation technologies have been used to increase the effectiveness of the active compounds using drug delivery systems in situations where water solubility is low and to improve long-term stability [22]. Of them, nanoliposomes (LPs) are spherical, single or multi-layered vesicles that can be micro- or nanosized but can trap both hydrophobic and hydrophilic compounds [23]. LPs are known as systems that provide and enhance the passage of active compounds both in the epidermis and in the deeper layers of the skin due to the similarity to cell membrane structure. Also, LPs are biologically compatible, biodegradable, non-immunogenic, and nontoxic systems that are widely used in cosmeceuticals [24,25]. 

For hundreds of years, natural ingredients have been used mainly for antioxidant, antimicrobial, and enzyme inhibitory activities for skin care and wound healing [26]. For this purpose, we studied (1) the antioxidant activities with radical scavenging assays and inhibition of β-carotene/linoleic acid co-oxidation of *S. aramiensis* extracts, (2) inhibitory effects of the most antioxidant active extracts (70% methanol) on elastase and collagenase enzymes, (3) the chemical composition of the 70% methanol extract, (4) the preparation and characterization of novel liposomal formulation containing 70% methanol extract, and (5) toxicity of 70% methanol extract and liposomal formulation on L929 fibroblast cell line.

## 2. Materials and Methods 

### 2.1. Plant Sample and Reagents

*Salvia aramiensis* which was used for experimental studies was collected on 01.06.2016 from Hatay (Belen), Turkey. Herbarium sample of the plant is stored in Mustafa Kemal University Science and Literature Faculty Herbarium (Plant Collector no: 1864). All reagents and chromatographic standards were obtained from Sigma Chemical Company (St. Louis, MO, USA). 

### 2.2. Extraction Procedure

The dried whole aerial part of the plant material (stem, leaves, and flower) was powdered and divided into three parts. The first part (50 g) was extracted with 70% methanol (MeOH) and the second part (50 g) was extracted with 70% ethanol (EtOH) three times in a shaking water bath at 37 °C for 24 h. The resulting extracts were combined separately and concentrated under vacuum with a rotary evaporator (37–38 °C). Since traditional use is in the form of tea, 2 g of aerial plant material is brewed with 100 mL of boiling water and left to rest for 10 min. This 2% form of infusion was then lyophilized. Until the analysis time, lyophilized extracts were stored at −20 °C. 

### 2.3. Total Phenolic (TFC) and Flavonoid Content (TPC)

TFC was calculated as gallic acid equivalents (GAE) in mg/g dry weight of plant material [27]. TPC content was calculated as catechin (CA) equivalents in mg/g dry weight of plant material through the aluminum chloride colorimetric assay [28].

### 2.4. Antioxidant Activity

#### 2.4.1. 1,1-Diphenyl-2-Picrylhydrazyl Radical (DPPH^●^) Scavenging Activity 

The determination of DPPH^●^ radical scavenging was carried out by the method of Braca et al. [29]: 500 µL of sample (0.025, 0.05, 0.1, 0.15, 0.2, 0.3, 0.4, 0.6, 0.8, 1, 1.5, and 2 mg/mL) and standard rosmarinic acid (5, 10, 15, 20, 25, 30, 40, 50, 75, and 100 µg/mL) were added to 1.5 mL of a 0.1 mmol/L MeOH solution of DPPH. Percent inhibition was calculated using Equation (1) after reading the absorbance at 517 nm.

% inhibition = ((Abs_control_ − Abs_sample_)/Abs_control_) × 100 (1)

#### 2.4.2. 2,2’-Azino-bis(3-ethylbenzothiazoline-6-sulfonic acid (ABTS^+●^) Radical Scavenging Activity

The determination of ABTS^+●^ radical scavenging was performed as declared by Huang et al. [30]. The ABTS^●+^ radical (7 mM) and potassium persulfate (2.45 mM) were dissolved in water to a final concentration and left for 16 h in the dark at room temperature. Extracts and the standard (rosmarinic acid) were prepared at 50 and 100 µg/mL concentrations. Trolox was used as a reference standard; 0.1 mL of each sample was mixed with 3.9 mL ABTS^●+^ solution. After addition of each extract and standards, absorbances were read for 30 min at 734 nm each minute. The results are expressed as the Trolox equivalent antioxidant capacity (TEAC, mmol/L Trolox).

#### 2.4.3. Determination of Inhibition of β-Carotene/Linoleic Acid Co-Oxidation

Antioxidant activity of *S. aramiensis* extracts was evaluated according to the *β*-carotene bleaching method of Velioğlu et al. [31]. *β*-carotene (5 mg) was dissolved in chloroform (5 mL CHCl_3_), and then *β*-carotene solution (1.2 mL) was added to the flask containing linoleic acid and Tween 20. CHCl_3_ was removed by evaporation from the flask. Distilled water (300 mL) was quietly dropped to the mixture for emulsifying. The control solution was prepared by the same method without adding *β*-carotene. For autoxidation, the samples were set into a 50 °C water bath for 120 min and the blenching level was recorded every 15 min. Measurements were made at 470 nm for extracts, standards, and controls. Rosmarinic acid was used as a positive control. Antioxidant activity was calculated according to Equation (2).

AAC = (1 − (Abs^0^_sample_ − Abs^120^_sample_) / (Abs^0^_control_ − Abs^120^_control_)) × 100 (2)

### 2.5. Enzyme Inhibitory Activity

#### Anti-Collagenase (MMP-1) and Anti-Elastase Activities

The inhibitory effects of 70% MeOH extract of *S. aramiensis* on the collagenase and elastase enzymes, which cause collagen and elastin degradation and is induced by the formation of free radicals, were measured. The measurements were performed according to the kit procedures of Collagenase and Elastase Elisa kits (Sun Red Bio, China); 70% MeOH extracts were studied at 50, 100, and 200 µg/mL, and positive control rosmarinic acid was studied at 25 µg/mL concentration [17].

### 2.6. Qualitative and Quantitative Chromatographic Analysis 

#### 2.6.1. Analysis with Liquid Chromatography-Tandem Mass Spectrometry (LC-MS/MS) High Performance Liquid Chromatography (HPLC) Systems

LC-MS/MS analysis was performed with Absciex 3200 Q trap MS/MS detector. Experiments were carried out using Shimadzu 20A HPLC system coupled to an Applied Biosystems 3200 Q-Trap LC-MS/MS instrument equipped with an electrospray ionization (ESI) source operating in negative ion mode. A GL Science Intersil octadecyl silica gel (250 × 4.6 mm, i.d., 5 µm particle size) analytical column has been used for the chromatographic separation (at 40 °C). The flow rate of the solvent was set to 0.3 mL/min. Photo Diode Array (PDA) detector was used for detection. The elution gradient consists of mobile phases A and B and contains acetonitrile:water:formic acid. The ratios are 10:89:1–89:10:1, v/v/v, respectively. The composition of B was increased from 10% to 100% in 40 min. Collected data were handled by Analyst 1.6 software.

#### 2.6.2. Analysis with High Performance Liquid Chromatography (HPLC) Systems

HPLC experiments were performed with Agilent HP1100. Separations were performed reverse-phase Mediterranean-C18 analytical column (250 × 4.6 mm i.d., 5 µm particle size). At room temperature, flow rate was set to 1 mL/ min. Detection was carried out with PDA detector between the wavelengths of 200 and 550 nm. Three sets of solvent systems—methanol/water/acetic acid (10:88:2, v/v/v) (solvent A), methanol/water/acetic acid (90:8:2, v/v/v) (solvent B), and methanol (solvent C)—were used for elution. Injections were brought to equilibrium for 10 min. A complete confirmation of assay consisting of linearity, the lower limit of detection and quantitation (LOD and LOQ), intraday and interday accuracy, and precision of the method were performed. 

### 2.7. Liposomal Formulation Studies

#### 2.7.1. Preparation of Nanoliposomes

Dry film hydration method was used for preparing LPs; Dipalmitoylphosphatidylcholine (DPPC) and Cholesterol (Ch) were added in a 1:1 molar ratio to the flask. They were dissolved with methanol:chloroform (3:1 v/v) and evaporated under reduced pressure at ~ 42–44 °C using Heidolph Rotavapor. The dry film was hydrated by 1 mg/mL 70% MeOH extract of *S. aramiensis* and vortexed. After ultracentrifugation of LP suspension at 15,000 rpm at 4 °C for 60 min, the supernatant and LPs were separated [32].

#### 2.7.2. Determination of Nanoliposomes Characteristics

Zetasizer Nano ZS-Malvern was used to determine characterization parameters of LPs: Particle size (PS), zeta potential (ZP), and polydispersity index (PDI) were determined. After ultracentrifugation, the supernatant phase was analyzed by HPLC for determining the 70% MeOH extract contents of LPs. The types of LPs were monitored using scanning electron microscopy (SEM). 

#### 2.7.3. In vitro Release Study 

Release study was performed using Franz diffusion cells with a dialysis membrane (12,000 Dalton pore size) for 24 h at 37 °C. The donor compartment of the diffusion cells was filled with 70% MeOH extract-loaded LP suspension (2 mL); 2 mL phosphate buffer (pH 7.4) was added to the receptor compartment. At the end of 24 h, the released 70% MeOH extract amounts were analyzed by HPLC as described above.

### 2.8. Toxicity Assessment on L929 Cell Line

The L929 cell line (mouse fibroblast) was obtained from *American Type Culture Collection* (ATCC^®^ CCL-1™) Manassas, VA, USA). For L929 cells, 10% solution of Eagle’s Minimum Essential Medium (EMEM), inactivated horse serum, medium containing the antibiotic mixture, and L-glutamine were used. 

The toxicity of the 70% MeOH extract and 70% MeOH extract-loaded liposomes on L929 cell line was determined by the Sulforhodamine B colorimetric (SRB) assay. Cells were counted and seeded in 96-well plates (1 × 10^4^ in 100 μL cells/well). The plates were then kept for 24 h before adding the samples. Stock solution extract was prepared at the concentration of 2 mg/mL in cell culture medium containing 0.5% DMSO (Dimethyl sulfoxide) and diluted to 7.81, 15.625, 31.25, 62.5, 125, 250, 500, and 1000 μg/mL concentrations. The control group was prepared with media containing 0.5% DMSO. After addition of diluted extracts and 70% MeOH extract-loaded liposomes (suspended formulation in 1mL cell culture medium), plates were incubated for 24 h at the same conditions; 10 mm unbuffered Tris-Base (100 μL) was added to solubilize the bound SRB, and the color density was measured at 540 nm with ELISA (Biotek Synergy HT). Results are shown as mean values of six measurements [33].

### 2.9. Statistical Analysis

The experimental data are shown as mean ± SD. SPSS software version 12.0 was used for statistical analysis. Analysis of variance was performed using ANOVA procedures. Significant differences were determined by *Tukey’s pairwise* and *Games–Howell* comparison test (*p* < 0.05). The *Levene* test was used to analyze the variance homogeneity of the groups. EC 50 values were calculated using nonlinear regression curves (Sigma Plot 2001 version 7.0, SPSS Inc., Chicago IL, USA).

## 3. Results and Discussion

In this study, in vitro antioxidant activities of *S. aramiensis* extracts prepared with 70% methanol, 70% ethanol, and 2% infusion were evaluated, and enzyme inhibitor activities of the active 70% methanol extract were investigated using various *in vitro* methods. In the study, 70% methanol extract, which was found active as a result of antioxidant activity tests, was loaded into the nanoliposome formulation, and the release of the extract was examined. Also, phytochemical examination of this active extract was determined using chromatographic techniques (LC-MS/MS, HPLC).

*S. aramiensis* extracts were found to be rich in TPC (198.07 ± 2.77) like other *Salvia* species [34,35]. Also, TFC content (141.76 ± 1.64) was found to be higher than *S. virgata* [36]. Results are given in Table 1.

The nitrogen-centered, stable radical DPPH^●^ was scavenged by the extracts at physiological pH, and the results were referred as the EC50 (µg/mL) value. According to Table 2, the EC50 value of 70% MeOH extract was found as 28.4 μg/mL. The activity of rosmarinic acid was statistically significant (*p* < 0.05) compared to the activities of the *S. aramiensis* extracts. Based on the results given in Table 2, all extracts and rosmarinic acid succeed in scavenging the ABTS^●+^ radical and no significant difference (*p* > 0.05) was found between the activity of 70% MeOH extract at the concentration of 100 µg/mL and the activity of rosmarinic acid at 50 µg/mL concentration. According to Tukey test results, the activity of rosmarinic acid was statistically significant (*p* < 0.05) compared to the 70% EtOH extract and the infusion. *Salvia* species have been proven by studies that they have strong antioxidant activity [34,35,36,37]. Therefore, our results have been found to be compatible with the literature.

**Figure 1:**
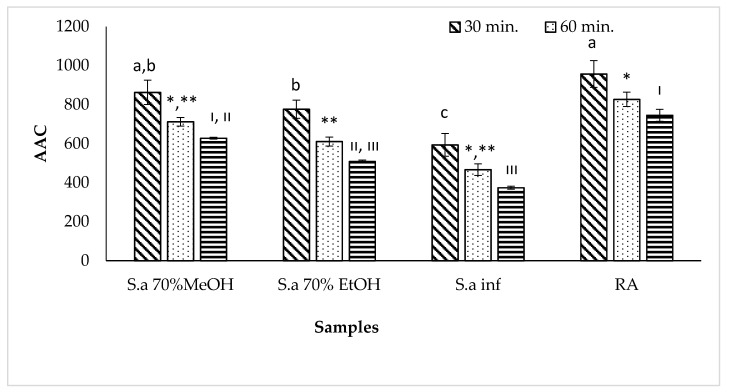
β carotene linoleic acid peroxidation activity of *S. aramiensis* extracts: Values = mean ± SD, statistical analyses by Games–Howell comparison test; bars with the same lowercase letters (a–d), same symbols (*, **) and Roman numerals (I, III) are not significantly (*p* > 0.05) different, *n* = 3. S.a 70% MeOH; *S. aramiensis* 70% methanol extract, S.a 70% EtOH; *S. aramiensis* 70% ethanol extract, S.a inf; *S. aramiensis* 2% infusion.

Oxidation of linoleic acid generates free radicals because the hydrogen atom is removed from the diallylic methylene groups of linoleic acid. These free radicals oxidize unsaturated β-carotene and degradation of the orange-colored β-carotene monitored by spectrophotometer [38]. However, the presence of *S. aramiensis* extracts showed an anti-bleaching effect of β- carotene. The AAC values of all extracts are given in Figure 1. According to the Levene statistic, no assumption was made for the variable (*p* < 0.001). According to the results of the Games–Howell test, the comments are as follows: The activities of rosmarinic acid and 70% MeOH extract are statistically the same (*p* > 0.05) and are statistically significant when compared with the activities of the remaining extracts (*p* < 0.05). In light of the results of three different antioxidant activity studies, it was decided to continue with 70% MeOH extract in the next part of the study. 

The results of the anti-collagenase and anti-elastase activities of the *S. aramiensis* 70% MeOH extract are shown in Table 3. According to the results presented in Table 3, there was no statistically significant difference between 100 and 200 µg/mL concentrated *S. aramiensis* extracts and rosmarinic acid which was used as the positive control (*p* > 0.05). The collagenase enzyme inhibition of the different concentrations of the *S. aramiensis* extract was found between 66.64%–72.66%. The anti-elastase activity of the extract obtained with different concentrations was statistically significant between each other and standard rosmarinic acid (*p* < 0.05). As can be seen in Table 3, 200 µg/mL concentrated *S. aramiensis* extract was found to be more active than rosmarinic acid and the inhibition percentage was found at 86.9%. The activities of 50 and 100 µg/mL concentrated *S. aramiensis* extracts and rosmarinic acid was found statistically the same (*p* > 0.05). Although there are studies on the wound-healing effect of *Salvia* species, the extracts do not have formulations prepared in nano-carrier [39,40,41].

**Figure 2:**
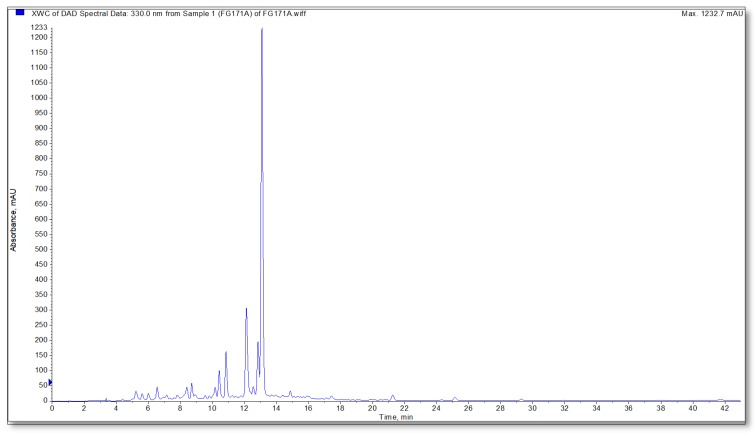
LC/MS/MS chromatogram of 70% MeOH extract of *S. aramiensis*.

The qualitative-quantitative analyses of the extracts were carried out using LC-MS/MS and HPLC systems and the results are presented in Table 4 and Table 5, respectively. In the present work, phenolic lignan, phenolic acids, and flavonoids were detected in negative ionization mode (Figure 2). At the beginning of the analysis, [M − H]^−^ ions at m/z 387 as well as fragment ions at m/z 207 and m/z 163 revealed the compound to be the phenolic lignin Icariside b5/medioresinol. The peak at Rt 8.7 showed an ion [M − H]^−^ at m/z 179 and a fragment peak at m/z 135. This compound was identified as caffeic acid. After the loss of a glucose unit (−162 Amu) from [M − H]^−^ ion at m/z 463, fragment ion [quercetin-H]– at m/z 301 was obtained, and this was identified as quercetin glucoside. At 10.7, a peak with m/z 447 fragmented to m/z 285 agreed with the fragmentation pattern of luteolin glucoside. With the presence of m/z 315 fragment ions obtained from the loss of 162 amu glucose moiety and the presence of fragment ion m/z 300 obtained by the loss of the methyl group from m/z 315, the peak (m/z 477) at 11.2 min was determined as the isorhamnetin glucoside. The peak at Rt 12.3 min had a molecular ion [[M − H]^−^ at m/z 717 and the base peak at m/z 519 with loss of 198 amu danshensu. The ions at m/z 537 [M-H-caffeic acid]−, m/z 475 [M − H-danshensu-CO_2_]^−^, and m/z 321 [M − H-danshensu-danshensu]− were seen. Based on the loss of danshensu and caffeic acid moieties, this compound was assigned as salvianolic acid E. The presence of salvianolic acid E before rosmarinic acid in the reverse phase column complies with the literature. The peak at 13.3 min was identified as rosmarinic acid. Rosmarinic acid showed a molecular ion [M − H]^−^ at m/z 359 and fragment ions m/z 197, 179, and 161. Medioresinol-O-glucuronide was observed at 14.9 min with a molecular ion at m/z 563. Medioresinol-O-glucuronide peak yielded a fragment ion at m/z 387 corresponding to the medioresinol moiety after the loss of glucuronide moiety. The peak at 17.3 min was tentatively determined as luteolin with m/z 285 and fragmented to m/z 151 and 133 which was consistent with the fragmentation pattern of luteolin. 

**Figure 3:**
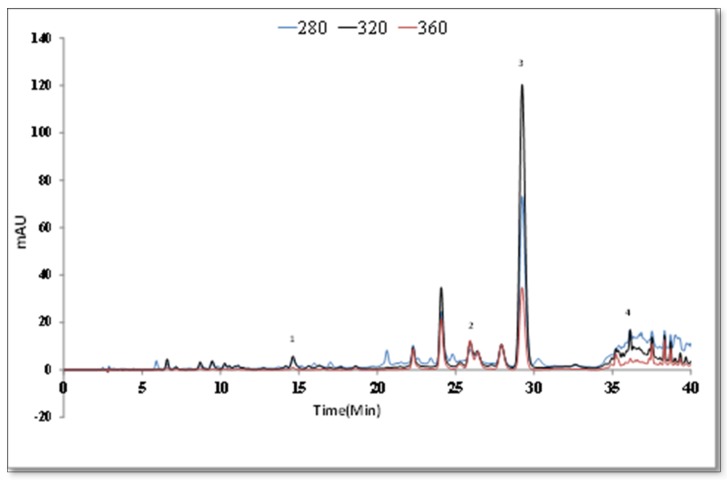
HPLC chromatogram of 70% MeOH extract of *S. aramiensis.*

The detection limit (LOD) and the quantitation limit (LOQ) were found as presented in Table 5 for the HPLC method when 10 μL was used as the injection sample volume. Comparing the retention times and UV spectra of the standards, caffeic acid, luteolin-7-O-glycoside, rosmarinic acid, and luteolin were determined (Figure 3). The peak increase of rosmarinic acid at 320 nm was linked to the maximum UV absorption at 329 nm of rosmarinic acid, a phenolic acid. The calibration curves of the standards are used to calculate the quantitative data. For *S. aramiensis*, the data obtained by both UV spectrophotometry and HPLC analysis showed that the 70% MeOH extract had a high phenolic content. Rosmarinic acid was the main compound, and its concentration was found as 122.32 ± 0.21 mg/g. The luteolin concentration of the extract was 4.06 ± 0.03 mg/g. 

**Figure 4:**
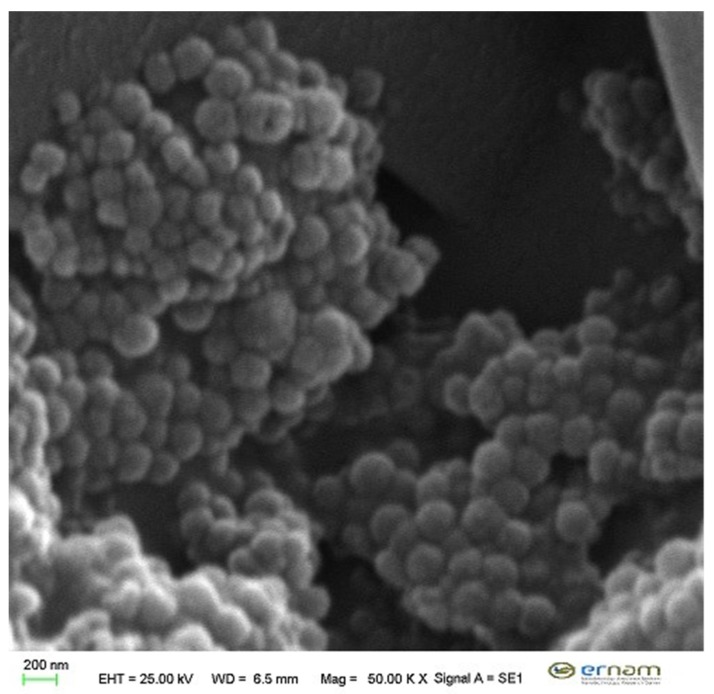
SEM image of liposome.

**Figure 5:**
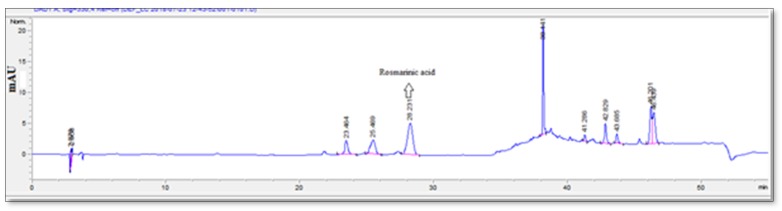
Chromatogram of released extract from liposome formulation.

*S. aramiensis* 70% MeOH extract-loaded LPs were prepared using dry film hydration. Nano drug delivery systems are systems that transmit the low-stability active substance to the lower layers of the skin; moreover, it offers the opportunity to work with a lower dose for a longer time. Therefore, liposomes were preferred. In the characterization studies, PS, ZP, and PDI of liposomes were found as 0.395 ± 0.004 µm, −35.2 ± 2.1 mV, and mono dispersed due to low PDI values (below 0.2) (Table 6). The encapsulating efficiency of the LPs was found as 13.83% ± 1.25%. In the field of pharmaceutical nanosystems, researchers have collected some information about size, stability, and morphology through the microscopic techniques. Of them, SEM was used in our study and the type and size of nanoliposomes that we developed in our study were determined by SEM. The liposomes, which are developed as seen in Figure 4, have a narrow particle size range and a smooth surface. In vitro release experiment from LPs was carried out for 24 h, and cumulative released amount of (RA) was found to be 60% ± 0.85% (83.7 µg/mL extract) with HPLC analyses (Figure 5). 

In literature, some studies with nanocarrier systems such as liposome, nanoparticles, etc. were developed with *Salvia* extracts. In the previous study, anti-neurotoxic efficacy of nanoparticles derived from *Salvia officinalis* extract against methylmercury-induced rats was evaluated. Sixty % ethanol extract of *Salvia officinalis* was associated with poly(lactic-co-glycolic acid) (PLGA) nanoparticles to enhance the antioxidant activity against mercury-induced neurotoxicity [47]. In another study, liposome oral liquid containing *Cordyceps sinensis*, *Astragali* L., and *Salvia miltiorrhiza*, which improved liver function, was prepared for the treatment of Hepatitis B and its efficacy was evaluated [48]. Aisha et al. [49] studied LPs which were prepared to contain *Orthosiphon stamineus* Benth. (Lamiaceae) (OS) ethanolic extract. Liposomes were prepared by the conventional film method with particle size and zeta potential of 152.5 ± 1.1 nm and −49.8 ± 1.0 mV, respectively. Release studies showed 94% ± 0.1% cumulative release in non-formulated extract and 62.4% ± 0.1% after 24 h at 37 °C in OS liposomes [49]. For these reasons, our characterization parameters seem to be compatible with the literature and successful when compared with OS-loaded LPs. In that study, the released extract showed improvement in DPPH^●^ scavenging effect. The results showed strong cleavage of DPPH^●^ both on formulated and unformulated extracts. However, a stronger effect with unformulated extract (EC50 = 32.4 ± 0.5 μg/mL) in connection with the high rate of release was found compared to OS LPs (EC50 = 23.5 ± 1.1 μg/mL) [49].

The amount of released extract compared to the activity experiments in the present study seems to be sufficient in all the experiments; 83.7 µg/mL of extract released from the LPs is well above the DPPH radical scavenging EC50 value. When we examine the results of the concentrations that we have tested in enzyme inhibition assays, it is clear that the extract released from the LPs will inhibit collagenase and elastase enzymes by more than 66.64% and 76.68%, respectively.

**Figure 6:**
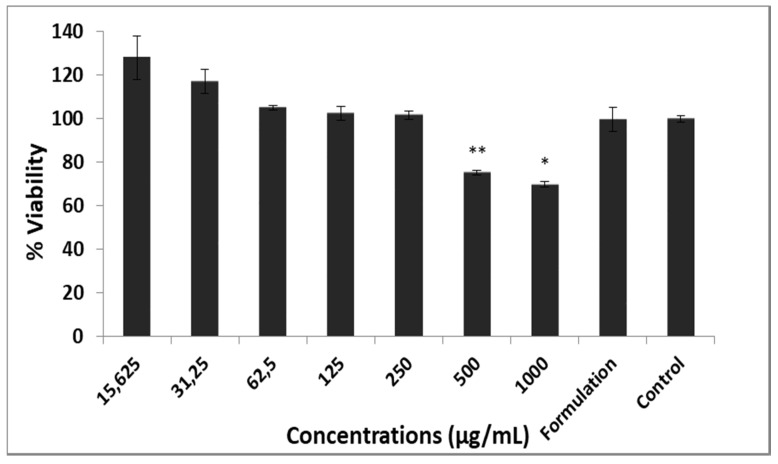
Toxicity effects of 70% MeOH extract and liposome formulation: Values given as mean ± SD were specified in the ±95% confidence interval (*n* = 3). Statistical analyses were by Games–Howell comparison test, and significant differences were reported as * *p* < 0.01; ** *p* < 0.05.

Fibroblast cells are commonly found in tendon, ligament, and skin tissues. They are generally described as collagen-producing cells and are known as the primary source of many extracellular matrix components [50]. L929 mouse fibroblast cells are frequently used to determine cytotoxic concentrations of many samples. It is also shown by international standards (ISO 10993 part 5, 1999; ISO 7405, 1997) as a reference cell for cytotoxicity testing [51]. The data acquired by the SRB cell viability method are given in Figure 6. Significance according to Levene statistics, homogeneity of variance, was found at *p* < 0.05 and Games–Howell test was used for statistical analysis. Between concentrations of 7.81–250 μg/mL, the 70% MeOH extract did not reduce the viability compared to the control group. The effect of 70% MeOH extract on cell viability at a concentration of 1000 μg/mL was 69%, and the influence of nanoformulation on cell viability was found to be 99.66%. Extract amounts obtained as a result of both encapsulation efficiency and release study are between 82.98 and 138.3 μg/mL. In the toxicity test, both concentrations were not toxic. The lack of toxicity of the LPs on the cell line is the first proof that it is reliable; also, it will shed light on future studies. This study is important in terms of proving the effectiveness of the nanoformulation containing extract rather than classical activity studies and proves the necessity of handling traditional plants with a modern understanding.

## 4. Conclusions

All data obtained from the study revealed the strong antioxidant effect of *S. aramiensis* as well as its potential wound-healing effect. The formulation study showed innovations in many respects as well as the potential of the extracts from plants to turn into products. Although the antioxidant activity of plant extracts is frequently studied, it is very important to show antioxidant capacities of formulated extracts. Therefore, in this study, *S. aramiensis* extract was first formulated with liposome, which is a nano-carrier system that attracts attention and is at the forefront with its advantages. With the successful demonstration of *in vivo* activity in future studies, a pharmaceutical product obtained from *S. aramiensis* will be safely functionalized.

## Figures and Tables

**Table 1:** Total phenol and flavonoid content of *S. aramiensis* extracts.

Extracts	Total Phenol (mgGAE/gextract) ^a^	Total Flavonoids (mgCA/gextract) ^b^
S.a 70% MeOH	198.07 ± 2.77	141.76 ± 1.64
S.a 70% EtOH	153.24 ± 0.94	93.28 ± 9.53
S.a inf	124.75 ± 1.58	118.85 ± 4.47

^a^ mgGAE/gextract: Total phenols expressed as gallic acid equivs milligrams of gallic acid per gram (dry weight) of extract. ^b^ mgCA/gextract: Total flavonoids expressed as rutin per gram (dry weight) of extract. Values are expressed as means ± standard error (*n* = 3). S.a 70% MeOH; *S. aramiensis* 70% methanol extract, S.a 70% EtOH; *S. aramiensis* 70% ethanol extract, S.a inf; *S. aramiensis* 2% infusion.

**Table 2:** DPPH^●^ and ABTS^●+^ radical scavenging activities of *S. aramiensis* extracts.

Extracts/Standard	DPPH● EC50 (µg/mL)	ABTS●+ (mmol/ L /Trolox)	
		50 µg/mL	100 µg/mL
S.a 70% MeOH	28.4 ± 0.002 ^b^	1.15 ± 0.101 ^a^	1.77 ± 0.09 ^b^
S.a 70% EtOH	36.6 ± 0.004 ^c^	1.59 ± 0.091 ^c^	1.65 ± 0.05 ^b,c^
S.a inf	32.6 ± 0.001 ^b^	1.24 ± 0.113 ^a^	1.37 ± 0.071 ^a,d^
Rosmarinic acid	4.4 ± 0.00 ^a^	2.16 ± 0.044 ^b,e^	2.56 ± 0.001 ^e^

Values = mean ± SD, statistical analyses by Tukey comparison test; values with the same lowercase letters (a–d) are not significantly (*p* > 0.05) different, *n* = 3. S.a 70% MeOH; *S. aramiensis* 70% methanol extract, S.a 70% EtOH; *S. aramiensis* 70% ethanol extract, S.a inf; *S. aramiensis* 2% infusion.

**Table 3:** Collagenase and elastase enzyme inhibition activities of *S. aramiensis* 70% MeOH extract and rosmarinic acid.

S.a 70% MeOH/Standard	Collagenase Enzyme Inhibition (%)	Elastase Enzyme Inhibition (%)
50 µg/mL	66.64 ± 1.53 ^b^	76.68 ± 1.21 ^a^
100 µg/mL	69.54 ± 1.49 ^a,b^	83.70 ± 1.72 ^b^
200 µg/mL	72.66 ± 0.70 ^a^	86.91 ± 0.81 ^c^
RA 25 µg/mL	72.89 ± 0.99 ^a^	80.58 ± 0.89 ^a,b^

Values = mean ± SD, statistical analyses by Tukey comparison test; values with the same lowercase letters (a–c) are not significantly (*p* > 0.05) different, *n* = 3. S.a 70% MeOH; *S. aramiensis* 70% methanol extract, RA: rosmarinic acid.

**Table 4:** LC-MS/MS results of 70% MeOH extract of *S. aramiensis*.

Rt	[M − H]^−^	Fragments	Identified Compounds	Ref
7.2	387	207, 163	Icariside b5/medioresinol	[42]
8.7	179	135	Caffeic acid	[43]
8.9	463	300, 271	Quercetin glucoside/hydroxyluteolin glucoside	[43]
10.7	447	285	Luteolin glucoside	[43]
11.2	477	315, 300, 285	Isorhamnetin hexoside	[44]
12.3	717	537, 519, 493, 321	Salvianolic acid E	[45]
13.3	359	197, 179, 161,	Rosmarinic acid	[43]
14.9	563	387, 207	Icariside b5/Medioresinol O-glucoronide	[46]
17.3	285	151, 133	Luteolin	[43]

Rt: Retention time; [M − H]^−^ molecular ion.

**Table 5:** HPLC results of *S. aramiensis* extract.

Compounds	Extract *	Standards		
	S.a 70% MeOH	Equation and r^2^	LOD	LOQ
Caffeic acid	1.84 ± 0.09	y = 44.283x + 40.553 r^2^ = 0.998	0.0067	0.0204
Luteolin-7-*o*-glucoside	3.70 ± 0.22	y = 39.422x + 78.434 r^2^ = 0.997	0.0085	0.0257
Rosmarinic acid	122.32 ± 0.21	y = 23.777x − 11.797 r^2^ = 0.997	0.2595	0.7865
Luteolin	4.06 ± 0.03	y = 41.981x − 70.752 r^2^ = 0.997	0.1407	0.4263

* Sa 70% MeOH: 70% MeOH extract. ^&^ mg/g_extract_, mean ± SD.

**Table 6:** Characterization of liposomes (*n* = 3).

Formulation	PS ± SD (µm)	ZP ± SD (mV)	PDI ± SD	EE ± SD (%)	RA ± SD (%)
Liposome	0.395 ± 0.004	−35.2 ± 2.1	0.107 ± 0.017	13.83 ± 1.25	60 ± 0.85

PS: particle size; ZP: zeta potential; PDI: polydispersity index; EE: encapsulation efficiency; RA: released amount of extract. Values are expressed as mean ± SD (*n* = 3).
